# Alterations of RNA Modification in Mouse Germ Cell-2 Spermatids Under Hypoxic Stress

**DOI:** 10.3389/fmolb.2022.871737

**Published:** 2022-06-14

**Authors:** Tong He, Huanping Guo, Lin Xia, Xipeng Shen, Yun Huang, Xiao Wu, Xuelin Jiang, Yinying Xu, Yi Tan, Yunfang Zhang, Dongmei Tan

**Affiliations:** ^1^ Laboratory Animal Center, Chongqing Medical University, Chongqing, China; ^2^ Medical Center of Hematology, Xinqiao Hospital, Army Medical University, Chongqing, China; ^3^ Clinical and Translational Research Center of Shanghai First Maternity and Infant Hospital, Shanghai Key Laboratory of Signaling and Disease Research, School of Life Sciences and Technology, Tongji University, Shanghai, China

**Keywords:** RNA modification, hypoxic stress, GC-2spd cells, spermatogenesis, high-throughput sequencing, mass spectrometry

## Abstract

Hypoxia is a known stress factor in mammals and has been shown to potentially impair male fertility, which manifests as spermatogenic dysfunction and decreased semen quality. Studies have shown that RNA modifications, the novel post-transcriptional regulators, are involved in spermatogenesis, and hypoxia-induced alterations in RNA modification in testes and sperm cells may be associated with impaired spermatogenesis in mice. However, the molecular mechanisms via which RNA modifications influence spermatogenesis under hypoxic stress conditions are unclear. In this study, we generated a mouse Germ Cell-2 spermatid (GC-2spd) hypoxia model by culturing cells in a 1% O_2_ incubator for 48 h or treating them with CoCl_2_ for 24 h. The hypoxia treatment significantly inhibited proliferation and induced apoptosis in GC-2spd cells. The RNA modification signatures of total RNAs (2 types) and differentially sized RNA fragments (7 types of approximately 80 nt-sized tRNAs; 9 types of 17–50 nt-sized sncRNAs) were altered, and tRNA stability was partially affected. Moreover, the expression profiles of sncRNAs, such as microRNAs, tsRNAs, rsRNAs, and ysRNAs, were significantly regulated, and this might be related to the alterations in RNA modification and subsequent transcriptomic changes. We comprehensively analyzed alterations in RNA modification signatures in total RNAs, tRNAs (approximately 80 nt), and small RNAs (17–50 nt) as well as the expression profiles of sncRNAs and transcriptomes in hypoxia-treated GC-2spd cells; our data suggested that RNA modifications may be involved in cellular responses under hypoxic stress conditions and could provide a basis for a better understanding of the molecular mechanisms underlying male infertility.

## 1 Introduction

Hypoxia is a stress factor that elicits widespread physiological responses; however, these responses may induce maladaptive consequences. Hypoxic stress has been reported to be responsible for altered male reproductive function (Jankovic and Stefanovic 2014). The findings of previous studies have suggested that in humans, hypoxic stress induces spermatogenic dysfunction (Okumura et al., 2003; Verratti et al., 2008), germ cell apoptosis (Reyes et al., 2012), and a decrease in semen quality (He et al., 2015) and sperm motility (Verratti et al., 2016). Moreover, hypoxia has been shown to significantly affect spermatogenesis in toads (Biswas et al., 1985), mice (Yin et al., 2018), and rats (Gosney 1984; Liao et al., 2010; Bai et al., 2018). Spermatogenesis is a dynamic and highly coordinated process based on the regulation of germ-cell proliferation, differentiation, and death in the testes (Singh and Jaiswal 2011). In a male infertility model subjected to hypobaric hypoxia treatment, hypoxia was found to induce spermatogenic cell (GC-2spd cells) apoptosis (Liao et al., 2010; Yin et al., 2018). Most studies on the molecular mechanisms underlying spermatogenetic defects have been focused at the DNA level. Recently, increased research attention has been directed toward the association between RNA molecules and fertility (Schuster et al., 2016), especially non-coding RNAs, such as long non-coding RNAs (lncRNAs) and short non-coding RNAs (sncRNAs), which have important regulatory functions in spermatogenesis. Previous studies have demonstrated that hypoxia promotes GC-2spd cell apoptosis via the miR-210-induced apoptotic signaling pathway (Lv et al., 2019). However, the RNA molecules involved in male reproductive injury under hypoxic stress conditions have not been clearly identified yet.

RNA modifications, which are newly discovered epitranscriptomic processes, play a crucial role in RNA structure formation, mRNA metabolism, mRNA translation, tRNA stability, and rRNA constitution during gene expression and tissue development (Roundtree et al., 2017; Sloan et al., 2017; Frye et al., 2018; Boo and Kim 2020). RNA modification has been reported to be significantly associated with fertility (Chen et al., 2016; Zhang J. et al., 2018). Among over 170 modified RNA nucleotide variants, RNA N6-methyladenine (m^6^A) was found to be dynamically regulated at different developmental stages of spermatogenesis in humans (Yang et al., 2016). Dynamic regulation of RNA modifications *in vivo* mainly depends on the activity of RNA methyltransferase/demethylase. The inactivation of the m^6^A methyltransferases, methyltransferase-like 3 (mettl3) and methyltransferase-like 14 (mettl14) may lead to spermatogonial stem cell depletion, inducing impairment in spermatogenesis (Lin et al., 2016; Lin et al., 2017). In addition, mutations in m^6^A demethylase alkB homolog 5 (ALKBH5) result in dysregulated spermatogenesis, low sperm count, poor sperm quality, impaired fertility (Zheng et al., 2013), and decreased testis size (Tang et al., 2018) in mice. Further investigation is required to determine whether other types of RNA modifications are associated with spermatogenesis. Moreover, as of now, the involvement of RNA modifications, including m^6^A, in spermatogenesis under hypoxic stress conditions is unknown. In this study, using our previously established high-throughput RNA modification detection platform and RNA-seq, we detected alterations in RNA modification profiles, tRNA stability, small non-coding RNA expression levels, and the transcriptome in GC-2spd cells exposed to hypoxic conditions, to investigate the molecular mechanisms via which RNA modifications influence spermatogenesis under hypoxic stress conditions. The results of the present study could enhance our understanding of the pathophysiology of male infertility.

## 2 Materials and Methods

### 2.1 Cell Culture

Germ cell-2 spermatids (GC-2spd) were purchased from the Conservation Genetics CAS Cell Bank (Shanghai, China) and cultured in DMEM (HyClone, Fisher Scientific International Inc., Logan, UT, United States) supplemented with 10% fetal bovine serum (Gibco, Thermo Fisher Scientific, Inc., Waltham, MA, United States) and 1% antibiotics (100 U/ml penicillin and 100 U/ml streptomycin, Invitrogen; Thermo Fisher, Waltham, MA, United States). GC-2spd cells were seeded into six-well plates at a concentration of 1 × 10^5^ cells/well (NEST, Wuxi, China) and incubated in an incubator containing 21% O_2_ and 5% CO_2_ (Thermo Fisher Scientific Inc., Waltham, MA, United States). The CoCl_2_-induced hypoxia cell model is commonly used in research to evaluate the effects of hypoxia on cell proliferation and apoptosis as well as other deleterious effects of hypoxia (Yu et al., 2018; Zhang and Chen 2018; Zhang et al., 2019). Here, after 24 h of culturing, cells to be subjected to hypoxia treatment were either cultivated in a medium supplemented with 150 μM CoCl_2_ (Sigma-Aldrich; Merck KGaA, Darmstadt, Germany) or transferred to a hypoxia workstation (Thermo Fisher Scientific Inc., Waltham, MA, United States) containing 1% O_2_, 5% CO_2_, and 94% N_2_ at 37°C. Experiments were routinely performed in triplicate (*n* = 3).

### 2.2 Proliferation Assay

GC-2spd cells were prepared as described previously under normoxic, hypoxic, and CoCl_2_ conditions. After 12, 24, 48, and 72 h of culturing, each set of cells was digested with trypsin and washed twice with phosphate-buffered saline (PBS, HyClone, Fisher Scientific International Inc., Logan, UT, United States) at room temperature. Cell counting was performed using an improved Neubauer hemocytometer (Qiujing Industrial Co., Ltd., Shanghai, China).

### 2.3 Apoptosis Assay

GC-2spd cells subjected to hypoxic treatment for 48 h or treated with CoCl_2_ for 24 h were stained using the PE Annexin V Apoptosis Detection Kit (BD Pharmingen; BD Biosciences, San Jose, CA, United States) according to the manufacturer’s protocol and analyzed using a flow cytometer and Kaluza software (Gallios; Beckman Coulter, Inc. Boulevard Brea, CA, United States).

### 2.4 Western Blotting

GC-2spd cells subjected to hypoxic treatment for 48 h or treated with CoCl_2_ for 24 h were washed twice with ice-cold PBS and then lysed in ice-cold radio immunoprecipitation assay lysis buffer (RIPA; Beyotime, Shanghai, China) supplemented with 1 mM phenylmethylsulfonyl fluoride (PMSF; Beyotime, Shanghai, China) for 0.5 h to extract total cellular proteins. Protein concentrations were determined using an enhanced BCA protein assay kit (Beyotime, Shanghai, China). A volume of 20 micrograms of protein was separated by 10% SDS-PAGE (Beyotime, Shanghai, China) and transferred onto nitrocellulose membranes (Millipore, Merck KGaA, Darmstadt, Germany). Next, the membranes were blocked with 5% non-fat dry milk in TBS containing 0.1% Tween (TBST) for 1 h at room temperature and then incubated overnight at 4°C with rabbit anti-HIF-1α antibodies (1:200; no. ab1; Abcam, Cambridge, United Kingdom) or mouse anti-β-actin antibodies (1:5,000; no. AF0003; Beyotime, Shanghai, China). Then, the membranes were washed with TBST and incubated with horseradish peroxidase-labeled goat anti-rabbit IgG (1:10,000, no. A0208, Beyotime, Shanghai, China) and horseradish peroxidase-labeled goat anti-mouse IgG (1:10,000, no. A0216; Beyotime) for 1 h at room temperature. The blots were visualized using an ultrasensitive enhanced chemiluminescence device (ECL, Thermo Fisher Scientific, Waltham, MA, United States). Band intensities were quantified using FluorChemHD2 software (ProteinSimple, San Jose, CA, United States).

### 2.5 Isolation of Total RNA and Specified Size RNA

Total RNA was extracted using TRIzol reagent (No. 15596026; Invitrogen; Thermo Fisher, Waltham, MA, United States) according to the manufacturer’s protocol. TRIzol (1 ml) was added to a microtube containing GC-2spd cells and vortexed vigorously. Then, the samples were incubated at room temperature for 5 min. Next, 200 μL of chloroform was added to the samples, vortexed for 15 s, incubated for 10 min at 20–22°C, and then centrifuged at 12,000 × g for 15 min at 4°C. The aqueous phase was collected in a microtube and mixed with an equal volume of isopropanol. After gentle mixing and incubation at −80°C for 30 min, the mixture was centrifuged at 12,000 × g for 15 min at 4°C. The final RNA pellets were resuspended in RNase-free water for the determination of RNA concentrations using a UV spectrometer.

Total RNA (3 μg) was separated by 7 M urea-denatured 15% PAGE and stained with the SYBR Gold solution (Invitrogen; Thermo Fisher, Waltham, MA, United States). Small RNAs with sizes of 17–50 nt and 80 nt were excised from the gel and extracted as previously described (Chen et al., 2016) for the quantification of RNA modification and for RNA-seq analysis.

### 2.6 Detection of Modified Nucleosides in RNA Molecules by LC-MS/MS

Purified RNA (50–100 ng) was transferred to a microtube and incubated with 5 U/ul alkaline phosphatase (no. P5521, Sigma Chemica; Merck KGaA, Darmstadt, Germany), 0.01 U/ul phosphodiesterase I (no. P3134; Sigma; Merck KGaA, Darmstadt, Germany), and 5 U/ul benzonase (No. E8263; Sigma; Merck KGaA, Darmstadt, Germany) at 37°C for 3 h. Then, 3 K devices with an omega membrane (Pall Corporation, New York, NY, United States) were used to remove the enzymes from the mixture by centrifugation. Mass spectrometric analysis was performed using a Xevo-TQ-S mass spectrometer connected to an Acquity-UPLC I-class system (Waters Corporation, Milford, MA, United States) equipped with an electrospray ionization source. Except for pseudouridine which was analyzed in the negative ion mode, for the analysis of the other molecules, the MS system was operated in the positive ion mode using a multiple reaction monitoring (MRM) scan model. LC-MS/MS data were processed using MassLynx software (version 4.1). The percentage of each modified ribonucleoside was normalized to the total amount of quantified ribonucleosides with the same nucleobase. For example, the percentage of m^6^A was calculated as the molar concentration of m^6^A/the molar concentrations of m^6^A+ Am + A+ m^1^A+ I + Im + m^1^I.

### 2.7 Analysis of tRNA Stability

Purified tRNA molecules (80 nt RNA fragments) were incubated at 37°C for 15 min in RPMI 1640-base systems: 15 ng of RNA in 9 μL of RPMI 1640 (HyClone, Fisher Scientific International Inc., Logan, UT, United States), 9 μL of RPMI 1640 containing 0.8% serum (FBS, Clark Bioscience, Virginia, United States), or 9 μL of RPMI 1640 containing 0.0003 μL of RNase A/T1 (2 mg/ml of RNase A and 5,000 U/ml of RNase T1). After incubation, the samples were immediately placed on ice, and RNA loading dye (2×, New England Biolabs Inc., Ipswich, United Kingdom) was added to them. A volume of four microliters of the samples was run on a 15% native PAGE gel at 4°C. The gels were stained with SYBR Gold before imaging.

### 2.8 Northern Blotting

Northern blotting was performed as previously described (Chen et al., 2016; Zhang J. et al., 2018). In brief, RNA extracted from GC-2spd cells was separated by 15% urea-PAGE, transferred onto nylon membranes (Roche, Basel, Switzerland), and cross-linked by UV at an energy level of 0.12 J. Then, the membranes were pre-hybridized with DIG pre-hybridization reagent (DIG Easy Hyb Granules, Roche, Basel, Switzerland) for at least 1 h at 42°C. For tRNA-Gly, tRNA-Ala, and 5.8S-rRNA detection, the membranes were incubated overnight at 42°C with 20 µM DIG-labeled oligonucleotide probes synthesized by Takara (Takara Bio Inc., Dalian, China), listed as follows:

tRNA-Gly: 5′-AAT​TCT​ACC​ACT​GAA​CCA​CCC​ATG​C-3’;
tRNA-Ala: 5′-CGC​TCT​ACC​ACT​GAG​CTA​CAC​CCC​C-3’;
5.8S- rRNA: 5′-TTC​TTC​ATC​GAC​GCA​CGA​GC-3’.

The membranes were sequentially washed with low-stringent buffer (2 × SSC with 0.1% (w/v) SDS), high-stringent buffer (0.1×SSC with 0.1% (w/v) SDS), and washing buffer (1×SSC) at 42°C. After washing, the membranes were incubated in 1 × blocking buffer (Roche, Basel, Switzerland) at room temperature for 3 h, and then anti-DIG antibodies (Anti-Digoxigenin-APFab fragments, Roche, Basel, Switzerland) were added to the blocking buffer at a ratio of 1:10,000 and incubated for an additional 1 h. The membranes were washed four times with DIG washing buffer (1× maleic acid buffer and 0.3% Tween-20) for 15 min each time, rinsed with DIG detection buffer (0.1 M Tris–HCl and 0.1M NaCl, PH:9.5) for 5 min, and then incubated with the CSPD reagent (Roche, Basel, Switzerland) in the dark for 20 min at 37°C before being imaged using a ChemiDoc imaging system (Bio-Rad, Hercules, CA, United States).

### 2.9 Small RNA Sequencing and Data Analysis

For each RNA library, more than 10 million reads (raw data) were generated using an Illumina HiSeq 2000 device (Bgi Technology Co., Ltd., Shenzhen, China). Small RNA library construction, library quality validation, and RNA sequencing were performed by Personalbio small RNA service (Shanghai, China), as described in our previous article (Zhang Y. et al., 2018). Clean reads were obtained and analyzed using SPOTS1.1 (small non-coding RNA annotation pipeline optimized for rRNA- and tRNA-derived small RNAs: https://github.com/junchaoshi/sports1.1) (Shi et al., 2018). Expression levels were normalized to reads per million (RPM).

### 2.10 Transcriptomic Data Processing and Analysis

Transcriptome sequencing was performed by Personalbio (Shanghai, China). In total RNA, mRNA with a poly-A structure was enriched using oligo (DT) magnetic beads. Then, the mRNA was randomly interrupted, connectors were added to it, and cDNA fragments were synthesized by reverse transcription, using RNA as a template for library construction. Finally, PCR amplification was performed to enrich library fragments. After RNA extraction, purification, and library construction, the samples were subjected to double pair-end (PE) sequencing using the Illumina NovaSeq sequencing platform.

The raw sequencing data were filtered using Cutadapt (von Hoyningen-Huene et al., 2019); then, high-quality sequence fragments were spliced using HISAT2 (Gangaraj and Rajesh 2020) and quantified using StringTie (Pertea et al., 2015). Differentially expressed genes were screened using the edgeR function in the R package (Yang et al., 2019); GO enrichment analysis was performed using the clusterProfiler function in the R package (Yu et al., 2012); GSEA enrichment analysis was performed using GSEA software (Subramanian et al., 2005). Gene sets were considered statistically enriched if the normalized *p*-value was less than 0.05.

### 2.11 Statistical Analysis

Data are presented as mean ± SEM, and each experiment was repeated at least three times. Statistical analyses were performed using GraphPad Prism 8 (GraphPad Software, San Diego, CA, United States). Data on proliferation, apoptosis, and RNA modifications in GC-2spd cells were analyzed by ordinary one-way ANOVA multiple comparisons or two-way ANOVA multiple comparisons; multiple comparisons were performed using Sidak’s or Dunnett’s test. Differences were considered statistically significant at *p* < 0.05.

## 3 Results

### 3.1 Cellular Responses and Alterations in RNA Modification Signatures in GC-2spd Cells Under Hypoxic Stress Conditions

**FIGURE 1:**
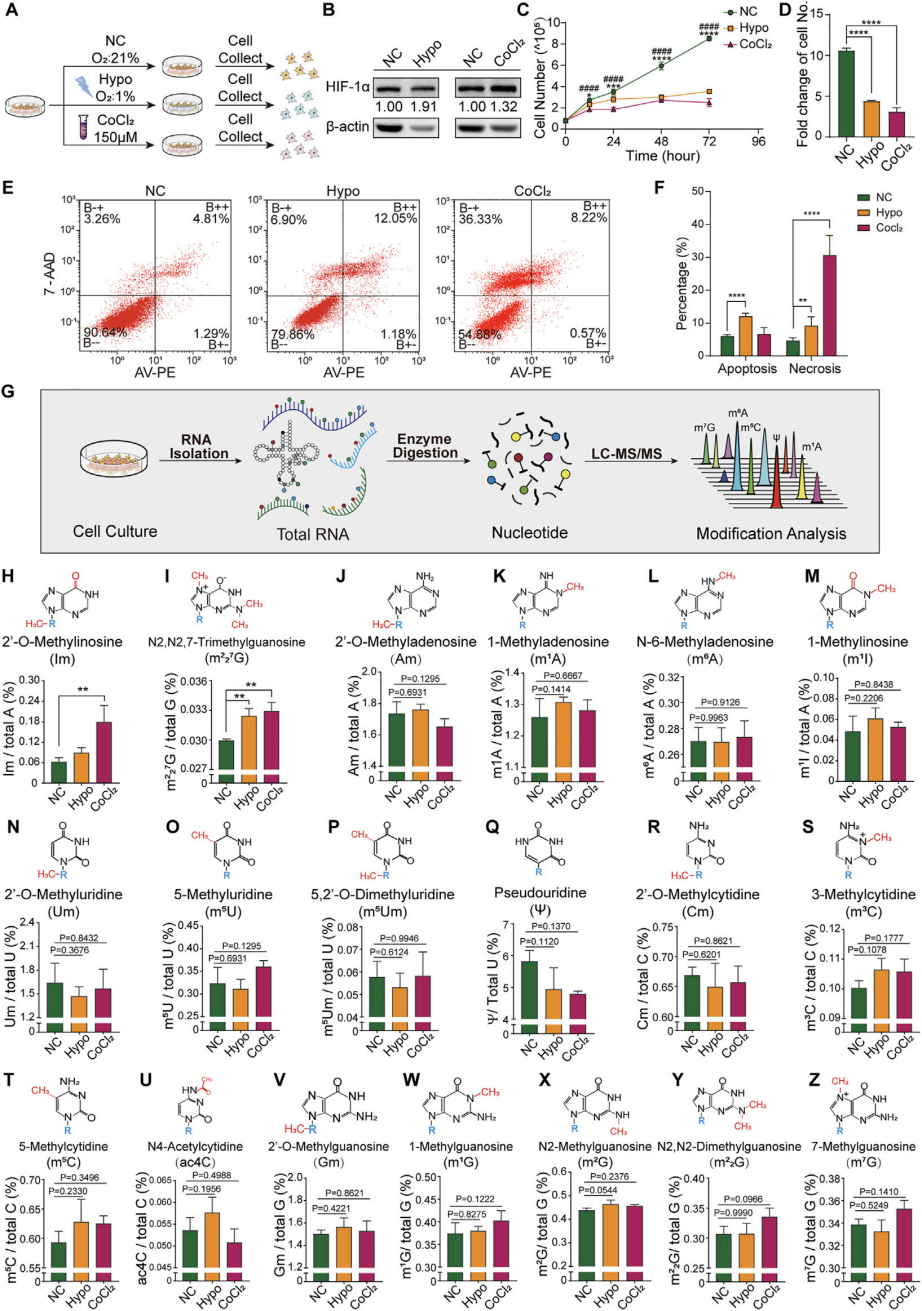
Cellular response and alterations in RNA modification signatures in GC-2spd cells subjected to hypoxic stress. **(A)** Schematic illustration of GC-2spd cell hypoxia treatment either by hypoxic culture (Oxygen Conc. 1%) for 48 h or normoxic culture in the presence of 150 μM CoCl_2_ for 24 h. **(B)** Western blot analysis of HIF-1α protein expression in GC-2spd cells exposed to hypoxia or CoCl_2_. **(C)** GC-2spd cell proliferation at 0, 12, 24, 48, and 72 h under normoxic, hypoxic, and CoCl_2_ treatment conditions. **(D)** GC-2spd cell growth rate at 72 h. **(E)** Flow cytometric analysis of apoptosis in GC-2spd cells subjected to hypoxia or CoCl_2_ treatment. **(F)** Statistical analysis of cell apoptosis and non-apoptotic cell death rates in GC-2spd cells. **(G)** Illustration of the detection of total RNA modifications in GC-2spd cells by LC-MS/MS. **(H–Z)** Relative levels of representative Im, m^2^_2_^7^G, Am, m^1^A, m^6^A, m^1^I, Um, m^5^U, m^5^Um, ψ, Cm, m^3^C, m^5^C, ac4C, Gm, m^1^G, m^2^G, m^2^_2_G, and m^7^G RNA modifications in GC-2spd cells subjected to hypoxia or CoCl_2_ treatment. Statistical analysis was performed by one-way analysis of variance (ANOVA), two-way ANOVA multiple comparisons, and Sidak’s or Dunnett’s multiple comparisons test. ^*^ *p* < 0.05, ^**^ *p* < 0.01, ^***^ *p* < 0.001, ^****^ *p* < 0.0001, ^####^ *p* < 0.0001 compared to NC. *n* = 3. Data in C, D, F, and H-Z are presented as mean ± SEM.

In our hypoxic GC-2spd cell model (Figure 1A), as compared to the normoxic group, there was a significant increase in HIF-1α expression levels in both the hypoxia and CoCl_2_ treatment groups (Figure 1B), indicating that the cells were in a hypoxic state. There was a decrease in the number of cells counted at 12, 24, 48, and 72 h following seeding (Figure 1C). At 72 h, the number of cells in the hypoxia and CoCl_2_ treatment groups was less than half of that in the normoxic group (Figure 1D). The percentages of apoptotic and non-apoptotic dead cells in the normoxic, hypoxia-treated, and CoCl_2_-treated groups were 5.96, 12.02, and 6.63, and 4.61, 9.15, and 30.63%, respectively. The high cell death rate in the CoCl_2_-treated group indicated the potential chemical toxicity of CoCl_2_ in GC-2spd cells (Figures 1E,F). To determine whether RNA modification was involved in cellular responses to hypoxic exposure, the total RNA modification profiles of GC-2spd cells were determined using our previously developed high-throughput LC-MS/MS-based RNA modification detection platform (Chen et al., 2016; Zhang J. et al., 2018) (Figure 1G). In total, 19 types of RNA modifications were detected and quantified, of which the levels of two (Im and m^2^_2_^7^G) were significantly increased in GC-2spd cells (Figures 1H,I); the levels of the majority of RNA modifications were unchanged (Figures 1J-Z).

### 3.2 Alterations in tRNA Modification Levels and Stability in GC-2spd Cells Under Hypoxic Stress

**FIGURE 2:**
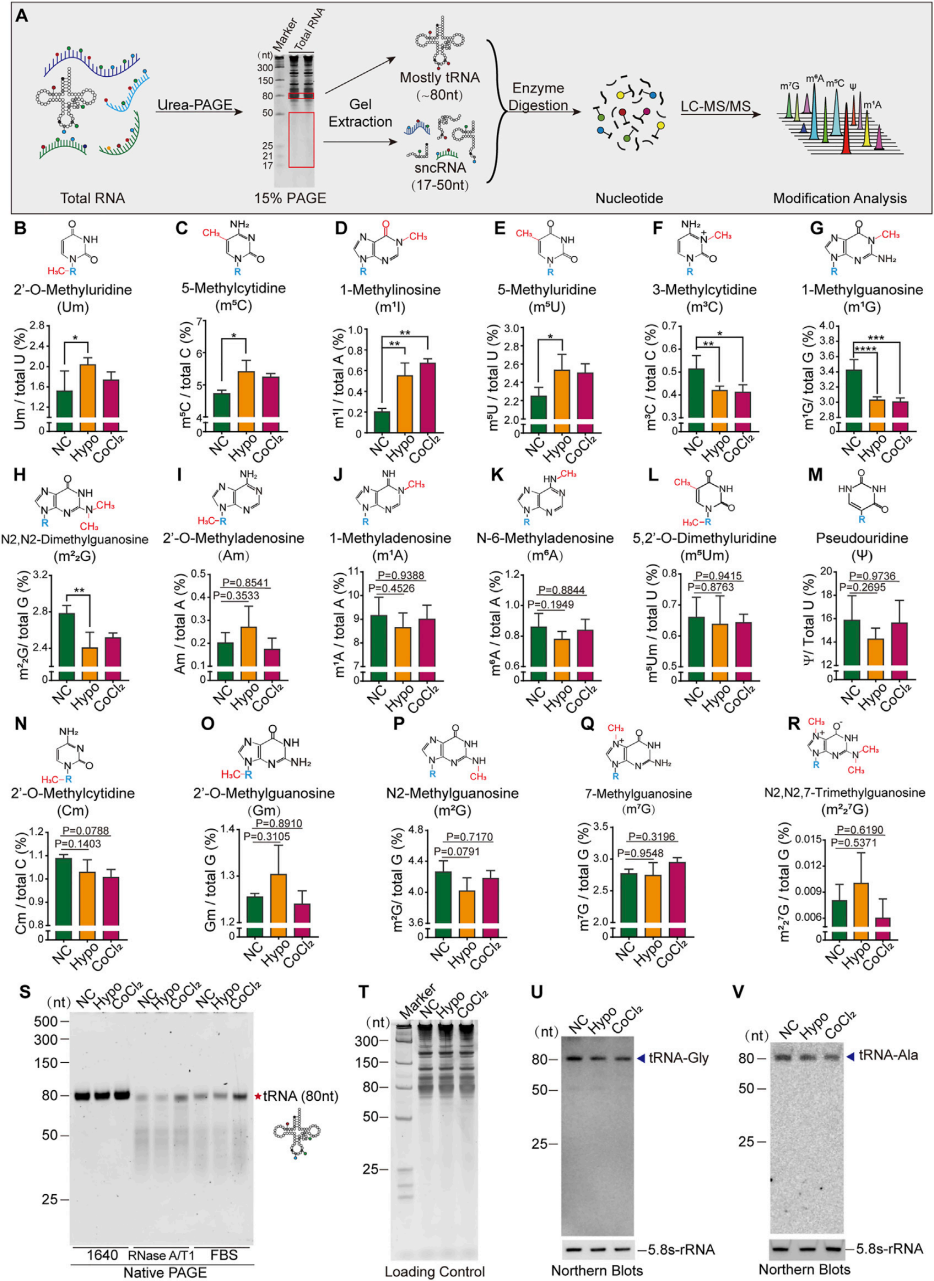
Cellular hypoxia treatment induced alterations in tRNA modification levels and tRNA stability in GC-2spd cells. **(A)** Illustration of the detection of RNA modifications in GC-2spd cells by LC-MS/MS. **(B–R)** Relative levels of Um, m^5^C, m^1^I, m^5^U, m^3^C, m^1^G, m^2^_2_G, Am, m^1^A, m^6^A, m^5^Um, ψ, Cm, Gm, m^2^G, m^7^G, and m^2^_2_^7^G in 80 nt-sized RNA fragments (mostly tRNA) in GC-2spd cells subjected to hypoxic conditions for 48 h or treated with 150 μM CoCl_2_ for 24 h. **(S)** Alterations in tRNA stability in GC-2spd cells subjected to hypoxic stress. Using a native PAGE gel, it was shown that the resilience of tRNA to degradation by RNase in the hypoxia- and CoCl_2_-treated groups was significantly different from that in the normoxia group, as determined using RNase A/T1 and 0.8% FBS (fetal bovine serum containing a unique combination of RNases). Each panel is presented as a representation of three independent experiments with similar results. **(T–V)** Representative tRNA expression levels were analyzed by Northern blotting. **(T)** Total GC-2spd cell RNAs were run on a 15% denatured PAGE gel presented as the loading control. **(U,V)** Expression levels of tRNA-Gly and tRNA-Ala (shown by arrow heads) in the normoxia, hypoxia, and CoCl_2_ groups; 5.8S-rRNA was used as the self-control. Blots are presented as a representation of three independent experiments with similar results. Statistical analysis was performed by one-way ANOVA and Dunnett’s multiple comparisons test. ^*^ *p* < 0.05, ^**^ *p* < 0.01, ^***^ *p* < 0.001, and ^****^ *p* < 0.0001, *n* = 3. Data in B–R are presented as mean ± SEM.

To further confirm the presence of alterations in tRNA and sncRNA modification in GC-2spd cells subjected to hypoxic treatment, the RNA modification profiles of RNA fragments of different sizes were determined by LC-MS/MS. Total RNA was first extracted and isolated by denatured PAGE, and then 80 nt-sized RNA molecules (most of them were tRNAs) and 17–50 nt-sized RNA molecules (sncRNA) were excised and purified for the identification of RNA modification signatures (Figure 2A). The levels of seven types of RNA modifications were altered in 80 nt-sized RNA fragments, with Um, m^5^C, m^1^I, and m^5^U being significantly upregulated, and the levels of m^3^C, m^1^G, and m^2^_2_G being significantly decreased following hypoxia and CoCl_2_ treatment (Figures 2B–H). Aside from these seven altered RNA modifications, the levels of 10 more types of RNA modifications in 80 nt-sized RNA molecules were unchanged (Figures 2I-R). It has been established that RNA modifications are closely associated with the structure and stability of tRNAs; therefore, tRNA stability was further evaluated by evaluating its resilience to RNase degradation in native PAGE gels. tRNA stability in the hypoxia and CoCl_2_ treatment groups was found to be significantly different from that in the normoxic group, as determined using RNase A/T1 and 20% FBS containing a unique combination of RNases (Figure 2S). Furthermore, the expression levels of representative tRNA, tRNA-Gly, and tRNA-Ala molecules were found to significantly decrease under hypoxic and CoCl_2_ treatment conditions (Figures 2T–V).

### 3.3 Alterations in RNA Modification Levels and the Expression Levels of Small Non-Coding RNAs in GC-2spd Cells Under Hypoxic Stress Conditions

**FIGURE 3:**
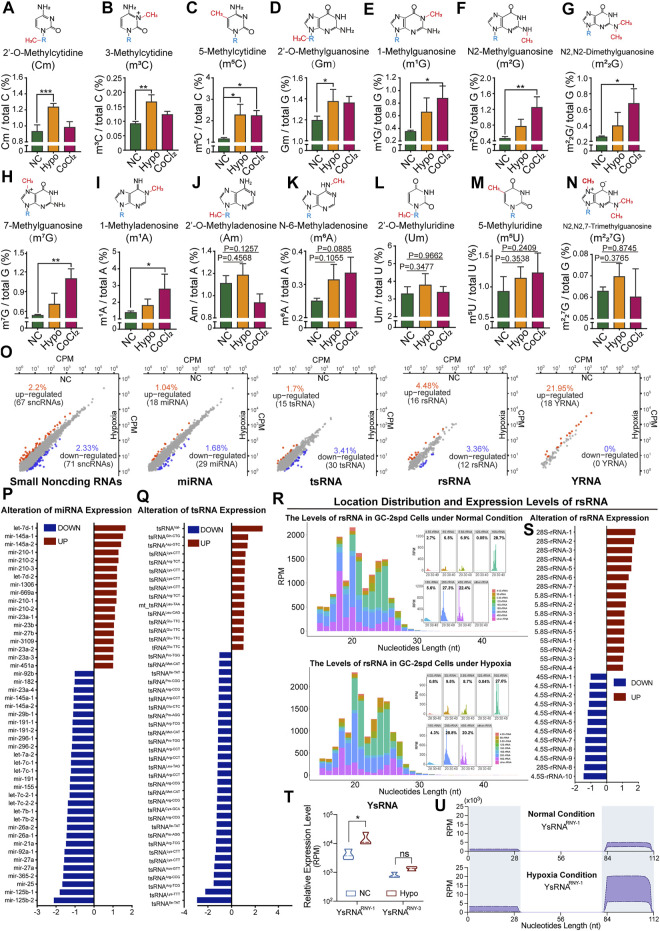
Alterations in RNA modification levels and the expression levels of small non-coding RNAs in GC-2spd cells subjected to hypoxic conditions. **(A–N)** Relative levels of Cm, m^3^C, m^5^C, Gm, m^1^G, m^2^G, m^2^_2_G, m^7^G, m^1^A, Am, m^6^A, Um, m^5^U, and m^2^_2_^7^G in 17–50 nt-sized RNA fragments in GC-2spd cells subjected to hypoxic treatment for 48 h or treated with 150 μM CoCl_2_ for 24 h **(O–U)** Sequencing analysis for differentially expressed small RNAs in GC-2spd cells subjected to hypoxic stress. **(O)** Alterations in four major small non-coding RNA (sncRNA) families in GC-2spd cells subjected to hypoxic stress: micro RNAs (miRNAs), tRNA-derived small RNAs (tsRNAs), rRNA-derived small RNAs (rsRNAs), and yRNA-derived small RNAs (ysRNAs). **(P)** Differentially expressed miRNAs. **(Q)** Differentially expressed tsRNAs. **(R)** Relative abundance and mapping locations of rsRNAs. **(S)** Differentially expressed rsRNAs. **(T,U)** Differentially expressed ysRNAs and their locations in GC-2spd cells subjected to hypoxic stress. Statistical analysis was performed by one-way ANOVA and Dunnett’s multiple comparisons test. ^*^ *p* < 0.05, ^**^ *p* < 0.01, and ^***^ *p* < 0.001, n = 3. Data in A–N are presented as mean ± SEM.

The RNA modification signatures of 80 nt-sized RNA molecules (most of which were tRNAs) were previously found to be altered and this affected tRNA stability in GC-2spd cells subjected to hypoxic stress. We thought it necessary to further investigate modifications and the expression levels of small RNAs (17–50 nt). The levels of nine types of RNA modifications (Cm, m^3^C, m^5^C, Gm, m^1^G, m^2^G, m^2^_2_G, m^7^G, and m^1^A) were significantly increased in treated GC-2spd cells (Figures 3A–I), while those of the other five types were unchanged (Figure 3J-N). High-throughput small RNA sequencing revealed alterations in the expression levels of 138 sncRNAs in cells subjected to hypoxic treatment (Figure 3O). More specifically, the expression levels of 18 miRNAs, including Let-7d, mir-145a, and mir-210, significantly increased, and the levels of 29 miRNAs, including mir-125b, mir-25, and mir-365, significantly decreased (Figure 3P). The levels of 15 tsRNAs (tRNA-derived small RNAs) were upregulated and those of 30 tsRNAs were downregulated, and this may partly have been due to RNA modification-induced alterations in tRNA stability (Figure 3Q). rRNA-derived small non-coding RNAs (rsRNAs) are newly identified small non-coding RNAs (Chu et al., 2017) that play important roles in paternally acquired epigenetic inheritance, sperm rapid response to metabolic alterations (Natt et al., 2019; Natt and Ost 2020), and organ inflammation (Chu et al., 2017). In this study, we also identified a large number of rsRNAs from 4.5, 5, 5.8, 12, 16, 18, 28, and 45S rRNAs. The most enriched rsRNAs were derived from 18, 28, and 45S rRNAs (Figure 3R). Under hypoxic conditions, there was an increase in the levels of most rsRNAs derived from 28, 5.8, and 5S rRNA, while the levels of those derived from 4.5S rRNA decreased, indicating that rRNA might be sensitive to hypoxic stress (Figure 3S). Moreover, the levels of YRNA-derived small RNAs (ysRNAs) were found to increase under cellular hypoxic stress conditions, especially those of small RNAs derived from RNY-1. The locations of ysRNAs on RNY-1 were further evaluated, and most ysRNAs^RNY1^ were found to be derived from the 3′ end of RNY1. However, the levels of ysRNAs^RNY1^ derived from both the 5′ and 3’ ends of RNY1 increased under hypoxic conditions (Figures 3T–U).

### 3.4 Gene Transcriptional Changes in GC-2spd Cells Under Hypoxic Stress Conditions

**FIGURE 4:**
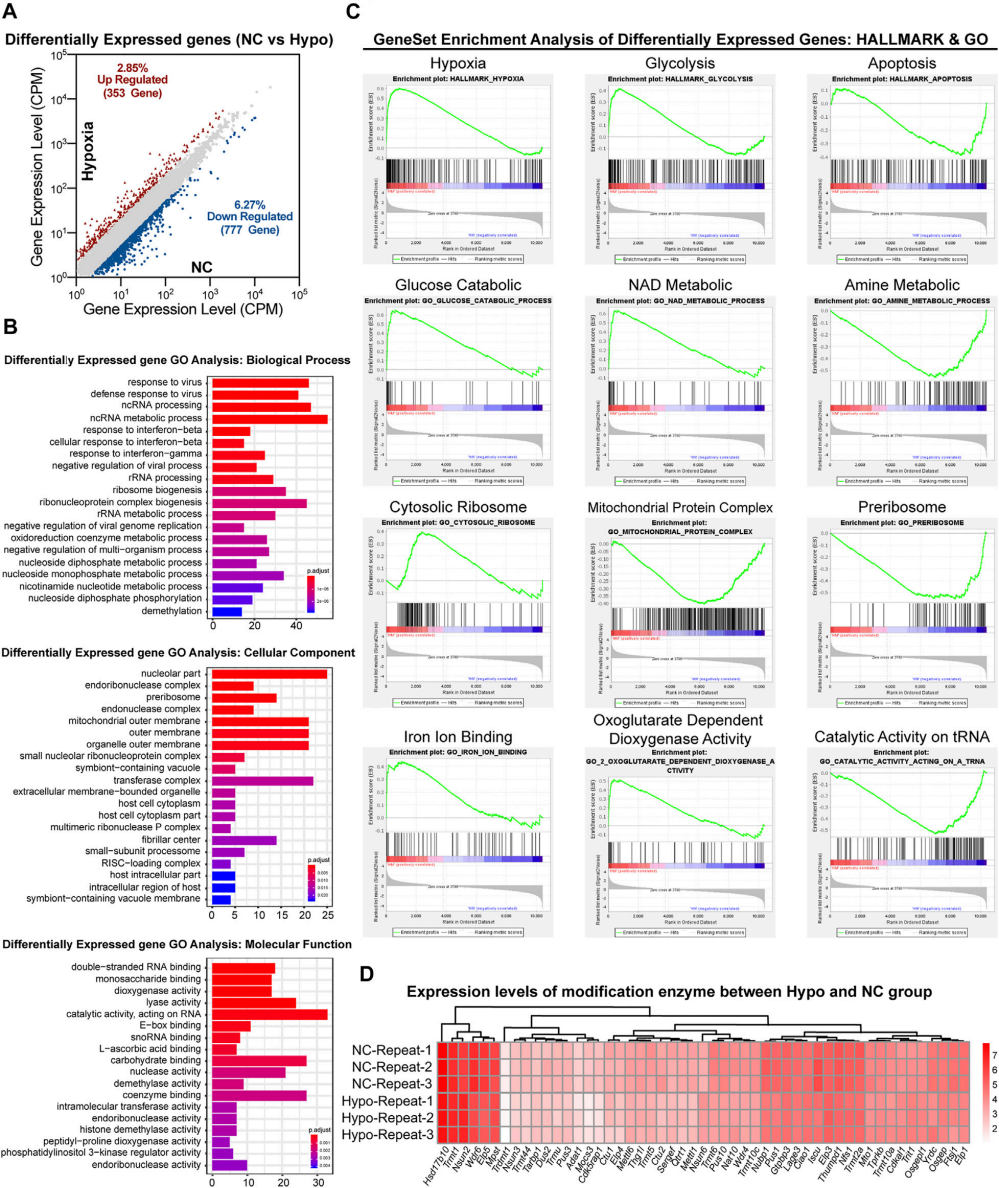
Transcriptomic analysis of gene expression in GC-2spd cells subjected to hypoxic stress. **(A)** Differentially expressed genes between the Hypo and NC groups. **(B)** GO analysis of differentially expressed genes between the Hypo and NC groups. The differentially expressed genes were enriched in the keywords “biological process,” “cellular component,” and “molecular function.” The *x*-axis represents the number of genes, and the color indicates the *p*-value (blue indicates a large *p*-value, and red indicates a small *p*-value). **(C)** GSEA analysis of differentially expressed genes in GC-2spd cells subjected to hypoxic stress in the GO and hallmark gene sets, respectively, ranked based on normalized *p*-value. **(D)** Heat map diagram showing the expression levels of modified enzymes in the Hypo and NC groups. Colors ranging from white to red indicate mRNA expression levels; the red color indicates high expression levels, while the white color indicates low expression levels.

As previously reported (Guzzi et al., 2018; Shi et al., 2019), modifications in sncRNAs, such as tsRNAs, are involved in the regulation of gene transcription. To evaluate the effects of RNA modifications on GC-2spd cell growth under hypoxic conditions via the regulation of gene transcription, transcriptomic sequencing of GC-2spd cells was conducted. A total of 1,130 genes were found to be upregulated (353 genes) and downregulated (777 genes) in the hypoxia-treated group (Figure 4A). Biological pathway analysis revealed that the dysregulated genes were enriched in RNA and nucleotide metabolic pathways. Cellular component analysis revealed that the differentially expressed genes were mainly located in the mitochondrial outer membrane, organelle membrane, and the transferase complex; molecular pathway analysis revealed that these altered genes were involved in RNA binding and acted on RNA and other molecular processes (Figure 4B). Gene set enrichment analysis showed that these differentially expressed genes were mainly enriched in aerobic respiratory pathways, including hypoxic, glycolytic, and apoptotic pathways (Figure 4C). Furthermore, RNA modification writers and erasers were also found to be regulated in the treatment groups as compared to the normal group, and this might be the direct reason why RNA modification levels were affected (Figure 4D).

## 4 Discussion

GC-2spd cells are immortalized cells derived from mouse spermatocytes that have been applied in research on the reproductive effects of toxicants, such as cigarette smoke (Esakky et al., 2014), cadmium (Seong et al., 2019), and di (2-ethylhexyl) phthalate (DEHP; (Fu et al., 2017)), and environmental factors, such as hypoxia (Zhou et al., 2018), fine particulate matter (PM_2.5_; (Zhang Y. et al., 2018)), and silica nanoparticles (SiNPs; (Ren et al., 2019)). Both the GC-2spd cell line and the spermatocytes isolated from mouse testes could be used to study early spermatogenesis. However, isolated spermatocytes are supposed to be of great value to represent the process *in vivo*. In fact, the spermatocytes obtained by the isolation technique are limited in number and stayed in different stages of division, while GC-2spd cells are easily available, stable, and homogeneous. Hypoxia-inducible factor 1α (HIF-1α) is a major hypoxia-induced transcriptional regulator that reflects the degree of oxygen deprivation (Iyer et al., 1998; Johnson et al., 2017). When cells are exposed to hypoxic conditions, HIF-1α rapidly accumulates (Jewell et al., 2001; Jankovic and Stefanovic 2014). In this study, both a hypoxic incubator and CoCl_2_ were used to establish hypoxic GC-2spd cell models. However, there was a less significant increase in HIF-1α expression levels in cells treated with CoCl_2_ than in those treated using the hypoxia incubator, and this could have resulted from differences in the degree of oxygen deprivation between these two groups.

Hypoxia has deleterious effects on the male reproductive system (Jankovic and Stefanovic 2014); however, its molecular mechanism is not fully understood. Our previous studies showed that hypoxia-induced alterations in RNA modification in mouse testes and sperm may be associated with impaired spermatogenesis (He et al., 2021). In this study, we used GC-2spd cells to investigate the potential mechanisms that altered modifications induced by hypoxia exposure could affect spermatogenesis. We found that hypoxia inhibited proliferation and induced apoptosis in GC-2spd cells, and this is consistent with the findings of a previous study (Yin et al., 2018). Alterations in RNA modification were also observed in hypoxia-treated GC-2spd cells, especially in tRNA and sncRNA, indicating that RNA modifications participated in the hypoxic stress response. Numerous RNA modifications occur in tRNAs (Pan 2018), and alterations of these modifications can affect the efficiency of endonuclease (Lyons et al., 2018), thereby affecting tsRNA biogenesis. We confirmed that alteration of RNA modification levels affected the tRNA stability, and the sncRNA profiles were largely changed by the hypoxia environment. There are more than 20 types of tRNA, and the isolation of individual tRNA needs a large number of total RNA; thus, we just analyzed the stability of tRNA-Gly and tRNA-Ala. tsRNA is derived from mature tRNA or tRNA precursors and participates in various biological functions (Kumar et al., 2016; Oberbauer and Schaefer 2018), such as stress responses (Schimmel 2018), the regulation of cell proliferation and apoptosis (Saikia et al., 2014), protein translation (Shi et al., 2019), ribosome biogenesis (Kim et al., 2017), and epigenetic inheritance (Chen et al., 2016; Zhang J. et al., 2018) through its RNA sequence and RNA modifications. Thus, the impaired spermatogenesis induced by hypoxia exposure (He et al., 2021) could be due to cell apoptosis regulated by abnormal expression of tsRNA or other sncRNA that are involved in regulation of gene expression (Lv et al., 2019). Subsequently, GO analysis and GSEA of transcriptomic data revealed that a large number of gene sets, which were mainly involved in hypoxic stress response and cellular aerobic respiration, as well as multiple related pathways, were altered. However, the target genes directly regulated by altered tsRNA and sncRNA remain unclear under hypoxia stress. Thus, further study is urgent to illustrate how the altered pathways are regulated by abnormal levels of RNA modifications and sncRNA.

The levels of seven types of RNA modifications in 80 nt-sized RNAs were found to be altered in hypoxia-treated GC-2spd cells. Among these, the levels of the m^5^C modification were upregulated. The presence of m^5^C in RNA has been shown to contribute to tRNA stability in mammals (Tuorto et al., 2012), and this may account for the observed changes in tRNA stability. Small RNA sequence analysis confirmed this finding, and forty-five types of tsRNAs were found to be regulated in GC-2spd cells exposed to hypoxic conditions. The levels of RNA modifications are commonly determined by the RNA modification writers and erasers as well as readers. The observed altered signature of RNA modifications in GC-2spd cells might suggest a differently expressed pattern of RNA modification regulators under hypoxia to adapt to the changing environment. For example, NOP2/Sun RNA methyltransferase 2 (Nsun2) and NOP2/Sun RNA methyltransferase 6 (Nsun6), the m^5^C RNA methylases, were upregulated under hypoxic stress conditions. Similarly, the level of m^5^C in tRNA was also increased. Previous studies have shown that Nsun2 (Tuorto et al., 2012) and Nsun6 (Haag et al., 2015) methylate tRNAs, and this is consistent with our findings. However, the levels of NOP2/Sun RNA methyltransferase 3 (Nsun3), another m^5^C RNA methyltransferase (Van Haute et al., 2016), did not increase, indicating that the increased m^5^C in tRNA was mainly determined by Nsun2 and Nsun6. Interestingly, there were significant differences in the types and levels of RNA modifications in 80 nt- or 17–50 nt-sized RNA fragments between the hypoxia- and CoCl_2_-treated groups. Small RNA sequence analysis revealed that the levels of a large number of sncRNAs, including miRNAs, rsRNAs, and ysRNAs, were also altered following hypoxia treatment, which might be accountable for the altered levels of RNA modifications in 17–50 nt-sized RNA fragments. We have not completely elucidated the exact mechanism that altered modifications under hypoxia exposure affected spermatogenesis. However, the altered RNA modification levels provided novel data for explaining hypoxia-induced GC-2spd cell impairment, providing direction for future research.

In summary, our data revealed hypoxia-induced alterations in RNA modification signatures in total RNA, 80 nt-sized RNA fragments (most of which are tRNA), and 17–50 nt-sized RNA fragments (sncRNA) in GC-2spd cells, and these might be responsible for changes in RNA stability and subsequent alterations in sncRNA expression profiles and the transcriptome under hypoxic conditions. Although the detailed mechanisms via which the alterations in RNA modification affect cellular responses to hypoxia stress need further research, data obtained in the present study offer a foundation for exploring the role of RNA modifications in the cellular responses of GC-2spd cells under hypoxic conditions and provide novel information on the molecular effects of hypoxia on male reproductive health.

## Data Availability

The authors acknowledge that the data presented in this study must be deposited and made publicly available in an acceptable repository, prior to publication. Frontiers cannot accept a article that does not adhere to our open data policies.
